# Receptor guanylyl cyclase-G is a novel thermosensor in Grueneberg ganglion neurons involved in coolness-induced ultrasonic distress calls in mice

**DOI:** 10.1186/2050-6511-14-S1-P14

**Published:** 2013-08-29

**Authors:** Ying-Chi Chao, Heinz Breer, Yuh-Charn Lin, Chih-Cheng Chen, Joerg Fleischer, Ruey-Bing Yang

**Affiliations:** 1 Institute of Biomedical Sciences, Academia Sinica, Taipei, Taiwan; 2 Institute of Physiology, University of Hohenheim, Stuttgart, Germany; 3 Institute of Pharmacology, School of Medicine, National Yang-Ming University, Taipei, Taiwan

## Background

In mammals, detection of ambient temperatures is mainly mediated by thermosensory neurons residing in the dorsal root ganglion (DRG) and trigeminal ganglion (TG) [1-3]. Recently, neurons in the Grueneberg ganglion (GG) of the murine nasal vestibule have been found to be activated by cool temperatures [4,5]. Unlike coolness-sensitive cells in the DRG and TG, neurons in the GG lack the TRPM8 channel [6] which is considered as a principal detector of cold [1-3]. Therefore, GG neurons are supposedly endowed with a so far unknown thermosensor. Interestingly, coolness-sensitive GG neurons express signaling elements associated with cyclic guanosine monophosphate (cGMP), including the cGMP-activated ion channel CNGA3 and receptor guanylyl cyclase-G (GC-G) [6-8]. Recent observations suggest that cGMP signaling is crucial for thermotransduction in the GG [8]. However, whether GC-G directly acts as a temperature sensor remains elusive.

## Materials and methods

A combination of biochemical and molecular biology methods, Ca^2+^ imaging as well as behavioural studies comparing wild-type and GC-G-knockout mice was used to elucidate the molecular and biological function of GC-G in sensing cool temperatures.

## Results

We show that GC-G is a thermosensory receptor that can be maximally stimulated by cool temperatures of about 15°C in both *in vivo* cellular cGMP accumulation assays and *in vitro* GC assays with a purified recombinant protein. Cells co-expressing GC-G and CNGA3 respond to cool temperatures via a rapid influx of calcium. Furthermore, we found a marked coolness-induced expression of the activity-dependent gene c-Fos in GG neurons of wild-type neonatal pups but not in GC-G-knockout conspecifics. Consistent with these findings, coolness-elicited ultrasonic vocalizations were significantly impaired in GC-G-knockout compared to wild-type pups.

## Conclusion

Our data suggest that GC-G is a novel thermosensory protein and that GG activation via GC-G by coolness is critical for the generation of ultrasound calls by isolated pups to elicit maternal care.
